# Late-season *Vibrio vulnificus* infection leading to purpura fulminans

**DOI:** 10.1016/j.jdcr.2025.04.041

**Published:** 2025-05-30

**Authors:** Joyce B. Kang, Kaylin Beiter, Travis Benson, Emily Hejazi, Andrew Friedman, Sofia Barrios, Sarah U. Kumar, Xiaowei Xu, Misha Rosenbach

**Affiliations:** a Harvard Medical School, Boston, Massachusetts; b Department of Dermatology, University of Pennsylvania, Philadelphia, Pennsylvania; c Department of Emergency Medicine, University of Pennsylvania, Philadelphia, Pennsylvania; d Department of Medicine, University of Pennsylvania, Philadelphia, Pennsylvania; e Department of Anesthesiology & Critical Care, University of Pennsylvania, Philadelphia, Pennsylvania; f Department of Pathology and Laboratory Medicine, University of Pennsylvania, Philadelphia, Pennsylvania

**Keywords:** climate change, fulminant purpura, infection, inpatient dermatology, *Vibrio vulnificus*

## Late-season *Vibrio vulnificus* infection leading to purpura fulminans

Noncholera *Vibrio* species are gram-negative bacteria most often found in warm coastal environments, including seawater and estuaries.^1^ Approximately 80,000 cases of *Vibrio* infection (vibriosis) occur annually in the United States. The majority experience a self-limited gastroenteritis (most commonly caused by *V. paraheaemolyticus* and *V*. *alginolyticus*), but 100-250 cases of potentially life-threatening *V. vulnificus* cases occur annually, manifesting as skin and soft tissue infections (SSTIs) from open wound contact or primary septicemia from ingestion of contaminated shellfish.^2^ Individuals with underlying liver disease or immunocompromising conditions are at higher risk of serious infection. However, severe morbidity and mortality can occur in otherwise healthy individuals following raw oyster consumption or direct water exposure.^3^ The mortality rate of *V. vulnificus* is high—25% for SSTIs and greater than 50% for primary septicemia.^4^ Historically, *V. vulnificus* infections are most commonly reported during summer months because the bacteria replicates faster in warmer temperatures.^5^ We present here an uncharacteristically late-season case of *vulnificus* sepsis with associated purpura fulminans.

## Case report

In the fall, a female in her late 60s with alcohol-related cirrhosis was transferred from an outside hospital in profound rapidly progressive shock, presumed to be septic. Initially, she experienced shortness of breath, diarrhea, and unilateral lower leg swelling within 2 days of having eaten half a dozen raw oysters at a beachside city in New Jersey (located at approximately 40°N latitude). She denied swimming in the ocean.

**Fig 1:**
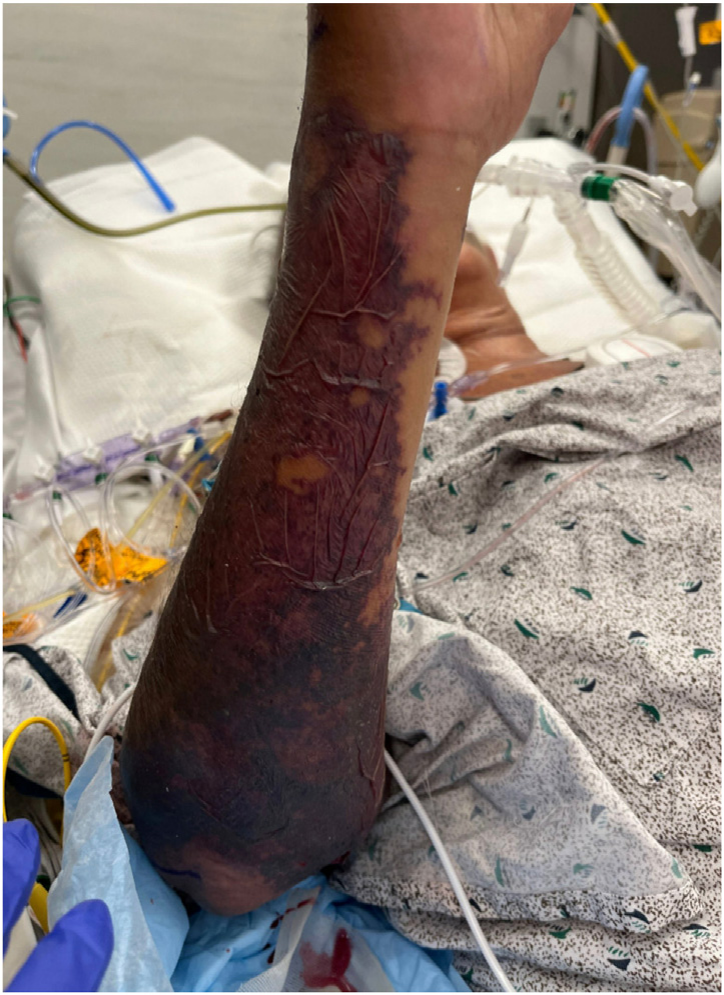
Arm on morning of admission. Exam showing retiform purpura and desquamation.

**Fig 2:**
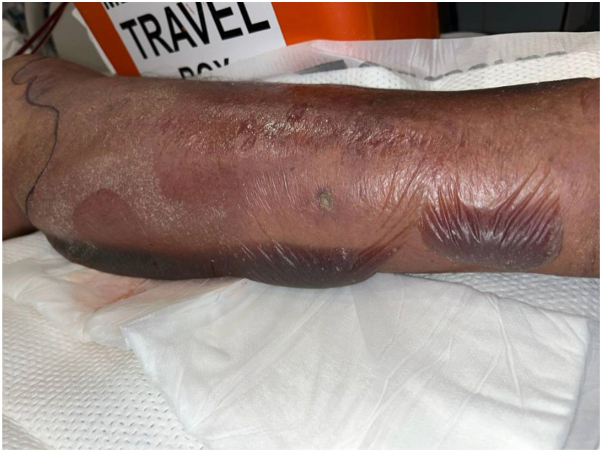
Leg on morning of admission. Exam showing erythema and rapidly progressing hemorrhagic bullae.

On admission to our hospital, her exam was significant for right upper extremity swelling with dark gray skin discoloration and diffuse nonpalpable purpura (Fig 1). Bullae appeared within an hour of admission and rapidly progressed to erosions. Similar changes were seen on the contralateral leg (Fig 2). Dermatology was consulted, and an urgent biopsy of skin was performed for pathology and tissue culture.

**Fig 3:**
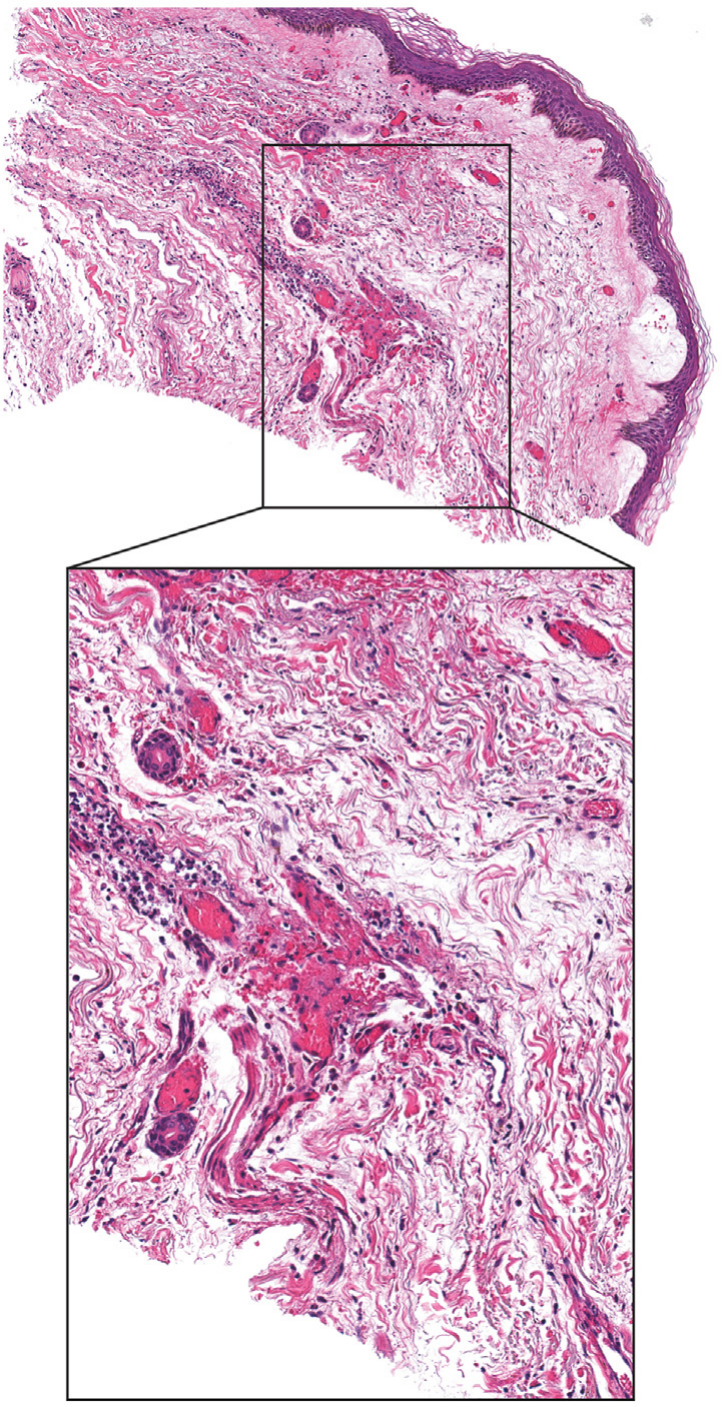
Histology. Hematoxylin and eosin-stained slide of punch biopsy taken from arm, showing extensive purpura with microthrombi in dermal vessels, interstitial acute and chronic inflammation, and papillary dermal edema and bullae formation. Inset shows higher power view of *black box* region.

At the outside hospital, the patient had been initially treated with broad-spectrum antimicrobials, including vancomycin, meropenem, doxycycline, and clindamycin for anti-toxin effect. Due to high suspicion for *V. vulnificus* infection, meropenem was stopped and she was switched to guideline-directed dual therapy with cefepime and levofloxacin. Histology from the skin biopsy demonstrated necrosis and dermal microthrombi (Fig 3), consistent with hypercoagulopathy; Grocott’s methenamine silver and gram stains were negative for microorganisms. Tissue culture did not grow any organisms, which may be expected as the patient was already on antibiotics at the time of biopsy. Blood culture data from the outside hospital and a culture from a bullae that was deroofed on the day she arrived at our hospital were both found to be positive for *V*. *vulnificus*.

The patient required intensive supportive care, with early aggressive intravenous fluid resuscitation, hemodynamic support with multiple vasopressors and inotropes, intubation and ventilator support, and continuous renal replacement therapy. She received stress-dose steroids and hydroxocobalamin for profound acidosis and distributive shock. Subsequent imaging showed extensive subcutaneous edema but no evidence of abscess. The patient’s liver function continued to decline and, unfortunately, she ultimately passed due to multisystem organ failure.

## Discussion

Purpura fulminans is a very rare complication of *Vibrio* infection, pathophysiologically attributed to widespread coagulopathy in the setting of bacterial endotoxin-mediated immune stimulation and coagulation-cascade dysregulation.^6^ While previous reports have documented *V. vulnificus* infection leading to purpura fulminans,^7^^,^^8^ our case is distinct in that it describes a late-season presentation at a mid-latitude (approximately 40°N), which is higher than Gulf Coast locations where *V. vulnificus* infections are most commonly reported.^2^ While it remains unclear whether the oysters the patient consumed were harvested from the Gulf of Mexico or mid-latitude waters, this case underscores the need for heightened clinical suspicion in regions beyond the traditional endemic zones.

Seasonal changes are known to influence *Vibrio* populations in the North Atlantic, with distinct year-round and warm-weather subgroups observed. The latter are believed to overwinter within sediment or zooplankton colonies, and overall population densities increase during warmer months.^7^ Both SSTIs from *V. vulnificus* and gastrointestinal disease from *V. paraheaemolyticus* have risen in tandem with warming environmental water temperatures.^1^^,^^9^, ^10^ This trend points to an alarming and durable shift in the habitats of clinically relevant *Vibrio* species.

The incidence of SSTIs caused by *V. vulnificus* has risen dramatically over the past 3 decades, with an associated shift northward in the uppermost geographic limit of cases.^1^ With models built on data from 1988 to 2018, Archer et al postulated that this increasing northward shift would be felt by 2041; our case not only corroborates this prediction, but also suggests an even faster progression than previously anticipated.

Dermatologists across the United States must be attuned to the impact of climate change on skin diseases, and remain an integral part of the hospital care team in order to assist in diagnosing rare, previously geographically-restricted conditions. Although historically concentrated in warm coastal waters, *V. vulnificus* is now a growing concern in mid-latitude environments. As demonstrated in our case, physicians should maintain a high index of suspicion for *V. vulnificus* infection in patients presenting with sepsis or rapidly progressing skin lesions, even outside traditional endemic regions. The northward expansion of *V. vulnificus* infections serves as a compelling example of how climate change can alter the epidemiology of serious infections and may serve as a sentinel for broader challenges for planetary health.

## Conflicts of interest

None disclosed.
